# Relationship between depression, anxiety, stress and smartphone addiction in COVID-19 nursing students 

**DOI:** 10.1590/1518-8345.6764.4056

**Published:** 2024-01-26

**Authors:** Marilyse de Oliveira Meneses, Elaine Maria Leite Rangel Andrade

**Affiliations:** 1 Universidade Federal do Piauí, Departamento de Enfermagem, Teresina, PI, Brazil.; 2 Scholarship holder at the Conselho Nacional de Desenvolvimento Científico e Tecnológico (CNPq), Brazil.

**Keywords:** Smartphone, Addictive Behavior, Nursing Students, Nursing, Anxiety, Depression, Teléfono Inteligente, Conducta Adictiva, Estudiantes de Enfermería, Enfermería, Ansiedad, Depresión, Smartphone, Comportamento Aditivo, Estudantes de Enfermagem, Enfermagem, Ansiedade, Depressão

## Abstract

**Objective:**

to verify the relationship between symptoms of depression, anxiety, stress and smartphone addiction in COVID-19 nursing students.

**Method:**

this was a descriptive-analytical study of 206 nursing students. A sociodemographic characterization and smartphone use instrument adapted from the literature and the following scales Depression, Anxiety and Stress Scale and Smartphone Addiction Inventory were used for data collection. Sociodemographic data and smartphone use were analyzed using descriptive statistics and the relationship between symptoms of depression, anxiety, stress and smartphone addiction was analyzed using multiple logistic regression.

**Results:**

the prevalence of smartphone addiction among nursing students was 129 (62.6%) and there was a relationship between symptoms of moderate depression (p=0.049), severe/very severe depression (p=0.005) and mild anxiety (p=0.028) and severe/very severe anxiety (p=0.019) and smartphone addiction.

**Conclusion:**

the data show that the construction and implementation of smartphone use policies in the academic and hospital context to prevent smartphone addiction and control associated risk factors is necessary.

## Resumen

**Objetivo:**

verificar la relación entre síntomas de depresión, ansiedad, estrés y dependencia del *smartphone* en estudiantes de enfermería durante la COVID-19.

**Método:**

estudio descriptivo-analítico realizado con 206 estudiantes de enfermería. Para la recogida de datos se utilizaron un instrumento de caracterización sociodemográfica y uso del *smartphone* adaptado de la literatura y las siguientes escalas: *Depression, Anxiety and Stress Scale* y *Smartphone Addiction Inventory*. Los datos sociodemográficos y el uso del *smartphone* se analizaron mediante estadísticas descriptivas y la relación entre síntomas de depresión, ansiedad, estrés y dependencia del *smartphone* se analizó mediante la regresión logística múltiple.

**Resultados:**

la prevalencia de dependencia del *smartphone* en estudiantes de enfermería fue de 129 (62,6%) y hubo relación entre síntomas de depresión moderada (p=0,049), grave/muy grave (p=0,005) y ansiedad leve (p=0,028) y grave/muy grave (p=0,019) y dependencia del *smartphone*.

**Conclusión:**

los datos revelan que la construcción e implementación de políticas de uso del *smartphone* en el contexto académico y hospitalario para la prevención de la dependencia del *smartphone* y el control de los factores de riesgo asociados son necesarias.

## Resumo

**Objetivo:**

verificar a relação entre sintomas de depressão, ansiedade, estresse e dependência de *smartphone* em estudantes de enfermagem na COVID-19.

**Método:**

estudo descritivo-analítico realizado com 206 estudantes de enfermagem. Para coleta de dados foram utilizados um instrumento de caracterização sociodemográfica e uso do *smartphone* adaptado da literatura e as seguintes escalas: *Depression, Anxiety and Stress Scale* e *Smartphone Addiction Inventory*. Os dados sociodemográficos e o uso do smartphone foram analisados por meio de estatísticas descritivas e a relação entre sintomas de depressão, ansiedade, estresse e dependência de smartphone foi analisada a partir de regressão logística múltipla.

**Resultados:**

a prevalência de dependência de *smartphone* em estudantes de enfermagem foi de 129 (62,6%) e houve relação entre sintomas de depressão moderada (p=0,049), grave/muito grave (p=0,005) e ansiedade leve (p=0,028) e grave/muito grave (p=0,019) e dependência de *smartphone*.

**Conclusão:**

os dados revelam que a construção e a implementação de políticas de uso do *smartphone* no contexto acadêmico e hospitalar para prevenção da dependência de *smartphone* e controle dos fatores de risco associados são necessárias.

**Highlights**

**(1)** High prevalence of smartphone addiction among nursing students.

**(2)** There was a relationship between symptoms of depression, anxiety and smartphone addiction.

**(3)** Nursing has a leading role in identifying and managing addictions.

**(4)** A multidisciplinary approach to the prevention and management of smartphone addiction.

**(5)** Smartphone addiction in nursing students is a new issue in Brazil.

## Introduction

Excessive smartphone use can cause dependence on this device, also known as “nomophobia” or an irrational fear of being without a mobile device ^(^ 1 ^)^. 

Smartphone addiction involves obsessive use of the mobile device, repetitive checking of messages or updates, tolerance or prolonged and intense use, withdrawal or feelings of agitation or distress and functional impairment or interference with other life activities and social relationships ^(^ 2 ^)^. 

Smartphone addiction is a problem that affects the physical and intellectual health of university students ^(^ 3 ^)^. In nursing students, the meta-analytical estimate of the prevalence of smartphone addiction was 22% in a sample of 2,780 individuals ^(^ 4 ^)^. In another survey of 298 university nursing students in the Northeast region of Brazil, the prevalence of smartphone addiction was 47.7% ^(^ 5 ^)^. These figures are worrying, both because of the prevalence rate and because of the consequences they can have on the daily lives of students with smartphone addiction ^(^ 4 ^)^. One of the reasons for concern is the distractions that the heavy use of cell phones can cause for university nursing students during clinical practice, reducing the quality of care and patient safety ^(^ 4 ^)^. 

Previous studies abroad have investigated the relationship between symptoms of anxiety, depression, stress and smartphone addiction in university nursing students. In Egypt, a study of 320 university nursing students found that the correlation between smartphone addiction and symptoms of depression (r = 0.996, p < 0.001) was statistically significant ^(^ 6 ^)^. An integrative review to identify the repercussions of smartphone use by university nursing students identified alarming levels of smartphone addiction that led to stress and anxiety and were reflected in the quality of sleep, learning and academic performance ^(^ 7 ^)^. In Korea, the results of another study of 421 university students indicated that anxiety and depression were related to smartphone addiction. According to the study, people with anxiety and depression can experience negative emotions in the real world, which leads them to compensatory behavior, seeking relief in the virtual world, which increases the possibility of smartphone addiction ^(^ 8 ^)^. 

In Brazil, there are few studies on smartphone addiction carried out with university nursing students ^(^ 5 ^)^. Since the translation and cultural adaptation of the Smartphone Addiction Inventory (SPAI) for Brazil ^(^ 9 ^)^, a study ^(^ 5 ^)^ found that alcohol use (p=0.036) and sleep quality (p<0.001) are related to smartphone addiction in nursing students. However, the relationship between symptoms of anxiety, depression and stress and smartphone addiction in university nursing students has not been investigated in the context of the COVID-19 pandemic. 

Due to the COVID-19 pandemic, numerous academic institutions around the world have been forced to close their doors and have adopted the use of online teaching and learning methods to provide the necessary material and try to save the academic year. This sudden leap in teaching methods has left students dissatisfied with their learning experience and caused stressful workloads that have led to more symptoms of depression and anxiety in university students ^(^ 10 ^)^. Perhaps the increase in these symptoms may have led university nursing students to greater smartphone dependence in the COVID-19 pandemic ^(^ 11 ^)^. 

The aim of this study was to verify the relationship between symptoms of depression, anxiety, stress and smartphone addiction in nursing students in the context of the COVID-19 pandemic.

## Method

### Study design

This is a descriptive-analytical study, written according to the STROBE tool (Strengthening the Reporting of Observational Studies in Epidemiology).

### Location and period

The study was carried out in the capital and countryside campuses of two public Higher Education Institutions (HEIs) in Piauí, Brazil: Federal University of Piauí (Teresina, Picos and Floriano campuses - UFPI) and State University of Piauí (Teresina, Picos and Floriano campuses - UESPI).

The researchers chose these HEIs intentionally, due to their ease of access, as well as the fact that they are well-established in the region, both because of the quality of their courses and their academic activities, and because of the results of the evaluations carried out by the Ministry of Education’s regulatory bodies.

### Population and criteria for selecting and defining the sample

The population consisted of nursing students (n=1145) from two public universities - UFPI (n=844) and UESPI (n=301). The sample was obtained by convenience and consisted of 206 nursing students who met the following inclusion criteria: (1) being 18 years old or older at the time of data collection, (2) having and using a smartphone, (3) having access to the internet via telephone. The exclusion criteria were: not completely filling in the items on the data collection instruments.

### Study instruments

Participants answered three instruments: sociodemographic characterization and smartphone use, adapted from the literature ^(^ 12 ^-^ 14 ^)^, Depression, Anxiety and Stress Scale (DASS-21) ^(^ 15 ^)^, and Smartphone Addiction Inventory (SPAI) ^(^ 9 ^)^. 

The instrument for sociodemographic characterization and smartphone use ^(^ 12 ^-^ 14 ^)^ consisted of six questions about age, gender and three more questions about smartphone use. 

The DASS-21 was developed in English ^(^ 15 ^)^ and adapted and validated for Brazil ^(^ 16 ^)^. It is a self-response scale made up of a set of three four-point Likert-type subscales that assess symptoms of depression, anxiety and stress. Each subscale is made up of seven items divided into three factors (Items Depression: 3, 5, 10, 13, 16, 17, 21; Anxiety: 2, 4, 7, 9, 15, 19, 20; Stress: 1, 6, 8, 11, 12, 14, 18). Each item has severity responses organized from zero (not applied at all) to three (applied a lot, or most of the time) ^(^ 15 ^)^. The result is obtained by adding up the answers to the items in each of the three sub-scales, which must be multiplied by two to calculate the final score and apply the cutoff, classified as: normal, mild, moderate, severe and very severe ^(^ 15 ^)^. The Cronbach’s alpha obtained for the Depression subscale was 0.92; for Stress it was 0.90 and 0.86 for Anxiety ^(^ 16 ^)^. 

SPAI was developed in Taiwan ^(^ 17 ^)^ based on Internet addiction screening questionnaires. Translated and culturally adapted for Brazil, it has 26 items subdivided into four categories: compulsive behavior, functional impairment, withdrawal syndrome and tolerance syndrome. It has at least nine positive responses as a cut-off point ^(^ 9 ^)^. The Cronbach’s alpha coefficient and the Kuder-Richardson coefficient of the SPAI-BR were both 0.887 ^(^ 9 ^)^. 

### Data collection

Data was collected remotely between April and July 2021 due to the COVID-19 pandemic. Initially, the researcher contacted the coordinators of the nursing courses to obtain the telephone number or email address of the class leaders of the nursing students at the universities. The class leaders were located, the objectives of the research were explained to them and the researcher requested that the invitation for the students to take part in the study be publicized in the class WhatsApp group, Instagram and email. The consent of the nursing students to take part in the study was obtained through the Informed Consent Form (ICF) (via Google Docs). A Google Forms link was then made available via WhatsApp, Instagram and email to fill in the data collection instruments.

### Data analysis

The data was analyzed using the Statistical Package for the Social Sciences (SPSS) version 22.0. Sociodemographic characteristics and smartphone use were analyzed using descriptive statistics, consisting of frequency and percentage for qualitative variables and mean and standard deviation for quantitative variables. The bivariate analysis of the variable’s depression, anxiety, stress and smartphone addiction was described using the Chi-square test and unadjusted odds ratio. The relationship was considered significant when P<0.05. The relationship between symptoms of depression, anxiety, stress and smartphone addiction was analyzed using a multiple logistic regression model. The variables were introduced into the models one by one (Stepwise Forward method). Variables with a p-value ≤ 0.05 remained in the final model. A significance level of 5% was adopted for all analyses.

### Ethical aspects

Before the study began, authorizations were obtained by email from the owners of the scales used in the research. The study was approved by the two public universities and by a Research Ethics Committee (protocol 4.688.110, on May 3, 2021). The nursing students were informed about the aim of the research and written consent was obtained from those who agreed to take part.

## Results

The majority of nursing students were female, 85.9% (n=177) and the average age was 21.7 years (SD 3.2) and median 21 (IR 20-23). The students used their smartphones an average of 7.9 hours a day (SD 3.7). It was found that 57.3% (n=118) students used their smartphone to access social networks, 34% (n=70) to work or study, 6.3% (n=13) to obtain information or news and 2.4% (n=5) to play games.

The prevalence of smartphone addiction among nursing students was 129 (62.6%). The prevalence of the categories that make up the SPAI: compulsive behavior, functional limitation, abstinence and tolerance is shown in Table 1. 

**Table 1 -:** Prevalence of items in the categories of the Smartphone Addiction Inventory (SPAI) scale answered by nursing undergraduates at public higher education institutions in Piauí (n = 206). Teresina, PI, Brazil, 2021

Variables	Yes n(%)
**Compulsive behavior**	
I feel willing to use the smartphone even when I feel tired	142(68.9)
I use my smartphone for longer and/or spend more money on it than I originally intended to	95(46.1)
Although smartphone use has had negative effects on my interpersonal relationships, the amount of time I spend on it remains the same	86(41.7)
I feel annoyed or down when I stop using the smartphone for a certain period of time	74(35.9)
I can’t control the urge to use the smartphone	87(42.2)
My leisure activities have decreased because of smartphone use	47(22.8)
My life would be dull if I didn’t have my smartphone	100(48.5)
Browsing on my smartphone has taken a toll on my physical health. For example, I use my smartphone when crossing the street, or while driving or waiting for something, and this use may have put me in danger	34(16.5)
I’ve been trying to spend less time using my smartphone, but I haven’t succeeded	82(39.8)
**Functional commitment**	
On more than one occasion, I’ve slept less than four hours because I was using my smartphone	82(39.8)
I feel more satisfied using my smartphone than spending time with my friends	28(13.6)
I feel pain or discomfort in my back, or discomfort in my eyes, due to excessive smartphone use	112(54.4)
Smartphone use has had a negative effect on my performance at school or work	66(32)
My interaction with my family has decreased because of my smartphone use	63(30.6)
My interaction with my family has decreased because of my smartphone use	63(30.6)
I’ve made smartphone use a habit and my quality and total sleep time have decreased	83(40.3)
I need to spend more and more time on the smartphone to achieve the same satisfaction as before	42(20.4)
I feel tired during the day due to late night/early morning smartphone use	68(33)
**Withdrawal syndrome**	
I feel uncomfortable/anxious/quiet when I don’t use my smartphone for a certain period of time	117(56.8)
I feel restless and irritable when I don’t have access to my smartphone	93(45.1)
The idea of using my smartphone is the first thing on my mind when I wake up in the morning	145(70.4)
I feel anxious or irritable when my smartphone is not available and I miss	
something when I stop using my smartphone for a certain period of time	86(41.7)
I feel a strong urge to use the smartphone again soon after I stop using it	105(51)
I can’t eat a meal without using my smartphone	70(34)
**Tolerance syndrome**	
I’ve been told more than once that I spend too much time on my smartphone	125(60.7)
I think I’ve been spending more and more time connected to my smartphone	159(77.2)
I have considerably increased the time I spend using my smartphone in the last 3 months	125(60.7)

The prevalence of moderate to extremely severe symptoms of depression, anxiety and stress among nursing students with smartphone dependence was 64.6%, 64.5% and 63.1%, respectively.

In the bivariate analysis, the level of moderate or severe/very severe depression (p<0.001), the level of mild (p=0.028), moderate (p=0.002) and severe/very severe anxiety (p<0.001), the level of mild (p=0.039), moderate (p=0.007) and severe/very severe stress (p<0.001) had a significant relationship with smartphone addiction. No significant relationship was found with the level of mild depression (p>0.05). After logistic regression, symptoms of moderate depression (p=0.049), severe/very severe depression (p=0.005) and mild anxiety (p=0.028) and severe/very severe anxiety (p=0.019) remained related to smartphone addiction ( Table 2). 

**Table 2 -:** Relationship between depression, anxiety, stress and smartphone addiction in nursing students at public higher education institutions in Piauí (n=206). Teresina, PI, Brazil, 2021

	Smartphone dependency			Crude odds (95%CI*)	p-value ^†^	Adjusted odds (95%CI)	p-value ^†^
	Yes n(%)	No n(%)	Total n(%)				
**Depression**							
Normal	29(39.7)	44(60.3)	73(100)	1	-	1	
Mild	13(46.4)	15(53.6)	28(100)	1.32(0.55-3.17)	0.541 ^‡^	0.78(0.29-2.12)	0,626 ^§^
Moderate	27(77.1)	8(22.9)	35(100)	5.12(2.05-12.82)	<0.001 ^‡^	2.84(1.00-8.06)	0,049 ^§^
Severe/very severe	60(85.7)	10(14.3)	70(100)	9.1(4.02-20.61)	<0.001 ^‡^	4.53(1.59-12.96)	0,005 ^§^
**Anxiety**							
Normal	26(35.6)	47(64.4)	73(100)	1	-	1	-
Mild	7(77.8)	2(22.2)	9(100)	6.33(1.22-32.71)	0.028 ^‡^	6.67(1.23-6.21)	0,028 ^§^
Moderate	21(70)	9(30)	30(100)	4.218(1.69-10.54)	0.002 ^‡^	2.64(0.95-7.33)	0,062 ^§^
Severe/very severe	75(79.8)	19(20.2)	94(100)	7.14(3.56-14.30)	<0.001 ^‡^	3.03(1.20-7.65)	0,019 ^§^
**Stress**							
Normal	30(39.5)	46(60.5)	76(100)	1	0.039 ^‡^	-	-
Mild	11(68.8)	5(31.3)	16(100)	3.37(1.07-10.68)	-	-	-
Moderate	22(68.8)	10(31.3)	32(100)	3.37(1.40-8.11)	0.007 ^‡^	-	-
Severe/very severe	66(80.5)	16(19.5)	82(100)	6.33(3.10-12.92)	<0.001 ^‡^		

^*^CI=Confidence Interval; ^†^p-value=Significance Level; ^‡^Chi-square test; § Logistic Regression

## Discussion

The results highlight the high prevalence of smartphone addiction among nursing students. In other studies, the prevalence of smartphone addiction was lower ^(^ 4 ^-^ 5 ^)^. Certainly, different prevalence rates may reflect differences arising from various local factors, including the relative availability and social acceptability of such technologies ^(^ 18 ^)^. In the COVID-19 pandemic, along with distance education, students’ smartphone and internet use habits have changed and their duration has been extended. However, the effect of this situation on problems that can develop due to heavy smartphone and internet use, such as nomophobia (fear of missing out), is still unknown ^(^ 19 ^)^. 

Logistic regression showed that there was a relationship between symptoms of moderate and severe/very severe depression and mild and severe/very severe anxiety and smartphone addiction. However, this relationship is complex and it is not yet known for sure whether symptoms of depression and anxiety increase among individuals with smartphone addiction ^(^ 20 ^)^, whether individuals with symptoms of depression and anxiety are more predisposed ^(^ 6 ^,^ 21 ^)^ or if there is a bidirectional correlation between symptoms of depression and anxiety and smartphone addiction ^(^ 22 ^)^. A study of university students in Serbia confirmed a two-way correlation between smartphone addiction and depression ^(^ 22 ^)^. 

The prevalence of moderate to extremely severe symptoms of depression, anxiety and stress among nursing students with smartphone addiction was 64.6%, 64.5% and 63.1%, respectively. The results of this study are similar to those of others which have also identified a relationship between symptoms of depression and smartphone addiction, and that it increases with levels of depression ^(^ 6 ^,^ 23 ^,^ 24 ^-^ 25 ^)^. In addition, the demands of smartphone use can predispose to depression due to stress ^(^ 6 ^)^. And depression, moodiness and nervousness are more frequent when people are offline ^(^ 26 ^)^. Other studies have also found a link between anxiety symptoms and smartphone addiction ^(^ 14 ^,^ 21 ^,^ 27 ^)^. 

In the last two years, due to the COVID-19 pandemic, there has been a significant increase in the time and intensity of use of electronic devices among university students and their relationship with various mental health problems ^(^ 28 ^,^ 29 ^-^ 30 ^)^. A survey of university students in Kazakhstan revealed a greater propensity to symptoms of depression and anxiety after the introduction of online learning ^(^ 31 ^)^. It is believed that online learning, the replacement of printed books with digital tools, free apps, platforms for videoconferencing and other electronic media have led students to use smartphones excessively ^(^ 32 ^)^ and the worsening of mental disorders ^(^ 33 ^)^. 

Systematic review with meta-analysis to measure the variation in the prevalence of major depressive disorder and anxiety before and during the COVID-19 pandemic estimated an additional 53.2 million cases of depressive disorder and 76.2 million (64.3 to 90.6) cases of anxiety disorders globally ^(^ 34 ^)^. Corroborating these findings, an online survey of 370 medical students showed that depressive symptoms were present in 78% of students and anxiety symptoms in 69% ^(^ 33 ^)^. In this study, smartphone use was significantly associated with the presence of depressive and anxiety symptoms ^(^ 33 ^)^. 

People who use smartphones excessively tend to feel more depressed and isolated without their cell phones, and may also experience other symptoms such as worry, tolerance, lack of control, withdrawal, mood modification, conflict, lying, overuse and loss of interest ^(^ 6 ^)^. Depression and anxiety are general reflections of psychological well-being, which are believed to be highly correlated with smartphone addiction ^(^ 33 ^)^. 

The results of this study should be weighed up against some limitations. Firstly, the sample was selected for convenience, which may affect the generalizability of the results. Secondly, self-report measures were used to collect information from the data collection instruments, so the results may have social desirability bias. Another possible bias of the study was the way smartphone addiction was measured among students, by answering questions on a scale rather than using objective, clinical measurement methods. It is recommended that future longitudinal or experimental studies be carried out to explore this information and provide a more accurate picture of the actual pattern of smartphone use and addiction among nursing students.

Considering the changes caused by the COVID-19 pandemic, including the introduction of online learning, advances in the development of mobile technologies, changes in social interactions and the important repercussions on the mental health of university students in the COVID-19 pandemic, this study is relevant because it provides information on the mental health of nursing students during the pandemic and addresses its relationship with smartphone addiction. The results found in this study contribute to the identification of risk factors for the problem and the construction of restrictive smartphone use policies in the academic and hospital environment that can prevent smartphone addiction in this target audience and control the related factors.

## Conclusion

There was a relationship between symptoms of moderate depression (p=0.049), severe/very severe depression (p=0.005) and mild anxiety (p=0.028) and severe/very severe anxiety (p=0.019) and smartphone dependence. These data provide specific information about the participants in this study in the context of the COVID-19 pandemic but may alert HEIs to formulate educational interventions to prevent and reduce factors related to mental health that can trigger or worsen smartphone addiction, seeking to reduce this problem mainly in the academic context. The preponderance of a multidisciplinary approach to the prevention and management of smartphone addiction is highlighted, with nursing playing a decisive role in recognizing and tracking aspects related to the physical and mental consequences of behavioral addictions and the basic principles for their management and treatment in an educational environment, as well as in healthcare settings.
